# MTiOpenScreen: a web server for structure-based virtual screening

**DOI:** 10.1093/nar/gkv306

**Published:** 2015-04-08

**Authors:** Céline M. Labbé, Julien Rey, David Lagorce, Marek Vavruša, Jérome Becot, Olivier Sperandio, Bruno O. Villoutreix, Pierre Tufféry, Maria A. Miteva

**Affiliations:** 1 Université Paris Diderot, Sorbonne Paris Cité, Molécules Thérapeutiques In Silico, INSERM UMR-S 973, Paris, France; 2 INSERM, U973, Paris, France; 3 RPBS, 75205 Paris, France

## Abstract

Open screening endeavors play and will play a key role to facilitate the identification of new bioactive compounds in order to foster innovation and to improve the effectiveness of chemical biology and drug discovery processes. In this line, we developed the new web server MTiOpenScreen dedicated to small molecule docking and virtual screening. It includes two services, MTiAutoDock and MTiOpenScreen, allowing performing docking into a user-defined binding site or blind docking using AutoDock 4.2 and automated virtual screening with AutoDock Vina. MTiOpenScreen provides valuable starting collections for screening, two in-house prepared drug-like chemical libraries containing 150 000 PubChem compounds: the Diverse-lib containing diverse molecules and the iPPI-lib enriched in molecules likely to inhibit protein–protein interactions. In addition, MTiOpenScreen offers users the possibility to screen up to 5000 small molecules selected outside our two libraries. The predicted binding poses and energies of up to 1000 top ranked ligands can be downloaded. In this way, MTiOpenScreen enables researchers to apply virtual screening using different chemical libraries on traditional or more challenging protein targets such as protein–protein interactions. The MTiOpenScreen web server is free and open to all users at http://bioserv.rpbs.univ-paris-diderot.fr/services/MTiOpenScreen/.

## INTRODUCTION

Drug discovery remains a very challenging endeavor and even the number of drugs approved by the FDA has been increased in 2014, it does not match the huge R&D investments (1). In order to foster innovation, several public–private partnerships have been established during these last 5 years (for instance the European Lead Factory, https://www.europeanleadfactory.eu/; IMI/, www.imi.europa.eu/). In this line, open screening initiatives play and will play a key role to facilitate the identification of new low molecular weight bioactive compounds that could be used as starting points for drug discovery or for chemical biology projects such as to investigate the importance of a target or of molecular mechanisms involved in diseases.

To date, many freely available online tools have been developed in that direction. For example, the widely used ZINC database (containing >13 million molecules) enables to rapidly identify drug-like molecules based on structural or pharmacophoric features (2). Several recent web servers performing binding site prediction (e.g. FPocket (3), GalaxySite (4), etc.), *de novo* drug design (e.g. e-LEAD3 (5)) or docking of a few ligands at time into a protein target (e.g. SwissDock (6), CovalentDock (7), etc.) have been reported. Yet, docking is not a trivial task and its performance strongly depends on the algorithms and scoring functions used, and on the definition of the binding site (8–13). Very few online structure-based virtual screening services have been reported thus far. The platform iScreen (14) performs virtual screening for over 20 000 traditional Chinese medicine compounds. DOCK Blaster (15) and the recently developed istar (16) perform large-scale screening using ZINC and protein–ligand docking.

Here we present the new web server MTiOpenScreen dedicated to small molecule docking and virtual screening. MTiOpenScreen offers users the possibility to screen in one run up to 5000 small molecules selected in different databases or up to 10 000 molecules selected among the 150 000 compounds ready to dock provided at MTIOpenScreen. The web server MTiOpenScreen includes two services, MTiAutoDock and MTiOpenScreen. MTiAutoDock allows to dock compounds into a binding site defined by the user or blind docking using AutoDock 4.2 (17) and MTiOpenScreen performs automated virtual screening using docking with AutoDock Vina (18). MTiOpenScreen is unique as providing original valuable starting collections for screening. Two in-house prepared drug-like chemical libraries containing compounds from the PubChem BioAssay Database (19) are provided for screening, one called Diverse-lib containing diverse molecules and the other, iPPI-lib, a collection enriched in putative inhibitors of protein–protein interactions (PPI). Thus, MTiOpenScreen enables researchers to run virtual screening computations on different chemical libraries ready for docking for traditional or more challenging protein targets like PPI, the latter representing a new class of promising therapeutic targets (20–23).

### THE MTiOpenScreen web server

**Figure 1.**
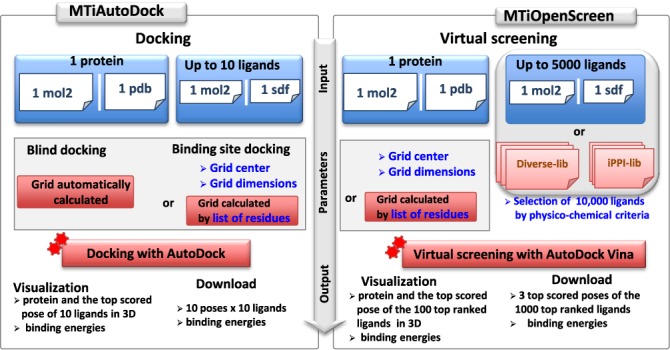
Workflow of MTiAutoDock and MTiOpenScreen. The user input is shown in blue. Calculations and chemical libraries provided by MTiOpenScreen are shown in red.

Figure 1 shows the overall workflow of MTiOpenScreen. The two tools MTiAutoDock and MTiOpenScreen are user friendly and suitable for non-advanced users. MTiAutoDock performs binding site docking or blind docking on the entire protein surface using AutoDock 4.2 (17) for up to 10 ligands uploaded by the user. Blind docking can identify putative druggable pockets at the protein surface as shown in the validation section. Virtual screening via MTiOpenScreen applies AutoDock Vina (18) and uses a gradient-based conformational search approach starting from the 3D structure of a protein target. User can screen up to 10 000 compounds of the Diverse-lib or iPPI-lib libraries. Users can apply physico-chemical filters to select 10 000 molecules belonging to a preferred chemical space. Otherwise user has the freedom to upload his/her own chemical library containing up to 5000 molecules that can be prepared by using the freely accessible web servers FAF-Drugs3 (24) for ADME-Tox filtering and Frog2 (25) for 3D conformation generation.

### Input

The protein structure can be uploaded in PDB or MOL2 format in MTiAutoDock and MTiOpenScreen. If the protein is provided in PDB, the structure is cleaned and preprocessed automatically: all HETATMs are removed and hydrogen atoms are added to the structure using MGLTools (17). Alternatively, users can prepare the protein structure manually and upload it in MOL2 format. If the protein is uploaded in MOL2 format, the structure will be used as is without any modification. If MOL2 format is used, all the solvent molecules should be removed and all hydrogen atoms should be added. Then, the user should define the binding site (except for blind docking) by providing the grid center and dimensions, or a list of residues defining the binding site.

For docking with MTiAutoDock, a unique 3D conformation with all hydrogen atoms added should be provided for up to 10 small organic molecules in a SDF or MOL2 file. For virtual screening with MTiOpenScreen, users can select one of the three options for the compound library:

- Ligands are uploaded by the user. For the time being, the number should not exceed 5000 molecules with a unique conformation in 3D with all hydrogen atoms added in a proper MOL2 or SDF format file. For correct docking results, the number of ligand atoms should not exceed 300 atoms.
- Up to 10 000 ligands can be taken randomly or selected according physico-chemical criteria chosen by the user from the prepared compound library Diverse-lib containing 99 288 diverse drug-like molecules.
- Up to 10 000 ligands can be taken randomly or selected according physico-chemical criteria chosen by the user from the ready to dock compound library iPPI-lib. This one contains 51 232 drug-like molecules with some specific shape and chemical properties likely to be efficient to inhibit PPIs.

### Output

MTiAutoDock and MTiOpenScreen provide several outputs. MTiAutoDock returns an interactive page allowing access to browse the 3D protein structure with the best-scored pose for each of the 10 docked ligands. The user can download the 10 best-scored poses for the 10 ligands docked by AutoDock. The predicted binding energies of the 10 best-scored poses for the 10 docked ligands can also be retrieved.

**Figure 2.**
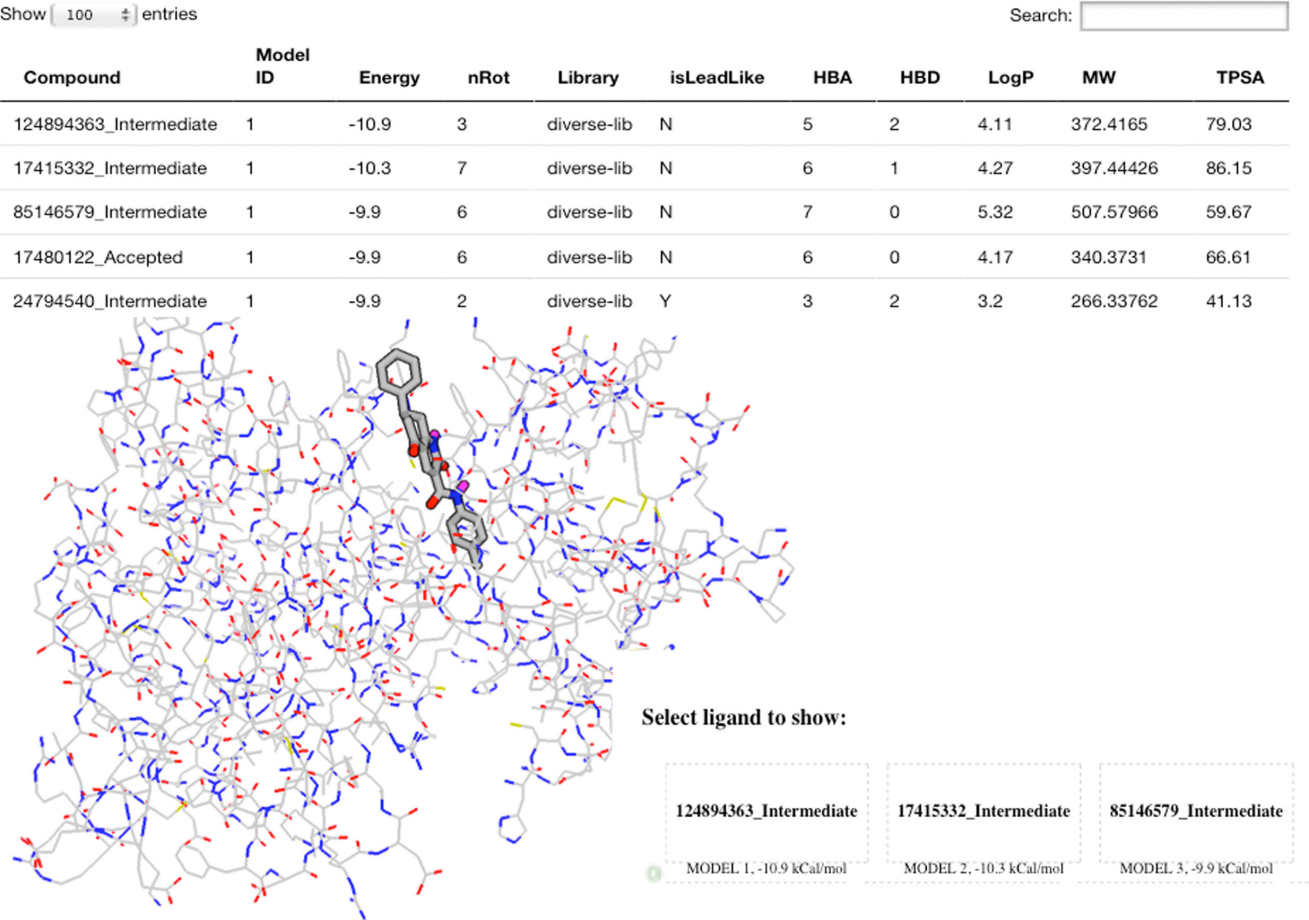
MTiOpenScreen interactive results pages. 3D protein structure of the VEGFR2 kinase domain (PDB ID: 4ag8) and the best docking pose are shown in 3D. The predicted binding energies of the top 100 ranked ligands and physico-chemical properties of ligands taken from Diverse-lib are also visualized. Ligand names in Diverse-lib and iPP-lib correspond to the PubChem SID. ‘Accepted’ annotation indicates that the compound does not contain toxic or PAINS groups. ‘Intermediate’ annotation indicates that the compound does not contain PAINS groups but contains toxicophores that belong to the low risk toxicity category (more information on low and high risk toxicity groups can be seen at http://fafdrugs2.mti.univ-paris-diderot.fr/groups.html).

MTiOpenScreen returns an interactive page allowing access to browse the 3D structure of protein with the best-scored pose of the 100 top ranked ligands (see Figure 2). The 3D visualization of the poses relies on a javascript Protein Viewer PV. The user can download the three best scored poses of the 1000 top ranked compounds among the 10 000 compounds screened with AutoDock Vina. The predicted binding energies of the three best-scored poses of the 1000 top ranked compounds and physico-chemical properties of ligands can also be retrieved. Thus, user can perform a thorough analysis of a large number of ligands among the 1000 top ranked compounds by using standalone programs like PyMOL (www.pymol.org/) or AutoDockTools (17) for a final selection of the best compound candidates.

### Performance of MTiOpenScreen

In order to validate our implementation of AutoDock and Vina software packages, we assessed the performance of MTiOpenScreen on several classes of important therapeutic protein targets: enzymes (kinases, serine protease, acetylcholinesterase), GPCR, nuclear receptors and PPI. Docking accuracy of blind and binding site docking with MTiAutoDock has been validated on 20 crystal structures of protein–drug complexes taken from the DrugPort database available at EMBL-EBI (http://www.ebi.ac.uk/thornton-srv/databases/drugport/). We have selected only complexes with high quality crystal structures verified using the software VHELIBS (26). In addition seven protein–ligand complexes with protein targets involved in PPI have been used for docking accuracy validation. Interestingly, the top scored pose of blind docking performed with MTiAutoDock identified the right binding pocket for 24 out of the 27 proteins assessed (results shown in Supplementary Figure S1 in Supplementary data). For 20 out of the 27 assessed proteins at least four poses among the 10 generated poses identified the right binding pocket. Binding site docking with MTiAutoDock also showed very good success rates (see Table 1): the top scored poses were found within an RMSD of 2.5 Å to the crystal structures for 60% of all tested complexes, and for 37% of them, the correct binding pose (RMSD < 1.5 Å) was found within the three most favorable ones. In order to assess the used 2 500 000 maximum number energy evaluations during the genetic algorithm search, we compared docking results of MTiAutoDock with 2 500 000 energy evaluations and docking with AutoDock4.2 with 25 000 000 energy evaluations (see Table 1). Overall, similar results were obtained. The crystal ligand poses were found within RMSDs of 2.5 Å in 82% and 78% of the time using 2 500 000 or 25 000 000 energy evaluations, respectively.

**Table 1.** Docking accuracy for 27 protein–ligand complexes using binding site docking with MTiAutoDock and 2 500 000 energy evaluations (GA1) and docking with AutoDock4.2 and 25 000 000 energy evaluations (GA2)

	All proteins		Factor X		Acetyl-cholinesterase		Kinases		GPCR		Nuclear receptors		PPI	
	GA1	GA2	GA1	GA2	GA1	GA2	GA1	GA2	GA1	GA2	GA1	GA2	GA1	GA2
The top scored pose RMSD < 1.5 Å	33.3%	29.6%	0%	50%	0%	0%	14.3%	14.3%	100%	100%	37.5%	37.5%	42.9%	28,6%
The top scored pose RMSD < 2.5 Å	59.3%	59.3%	100%	100%	0%	0%	28.6%	28.6%	100%	100%	75%	75%	71.4%	71.4%
The top three scored poses RMSD < 1.5 Å	37%	33.3%	50%	50%	50%	50%	14.3%	14.3%	100%	100%	37.5%	37.5%	42.9%	28.6%
The three top scored poses RMSD < 2.5 Å	66.7%	66.7%	100%	100%	50%	50%	28.6%	42.9%	100%	100%	75%	75%	85.7%	71.4%
Best RMSD < 1.5 Å	44.4%	37%	50%	50%	50%	50%	14.3%	14.3%	100%	100%	37.5%	37.5%	71.4%	42.9%
Best RMSD < 2.5 Å	81.5%	77.8%	100%	100%	50%	50%	42.9%	42.9%	100%	100%	100%	100%	100%	85.7%

**Figure 3.**
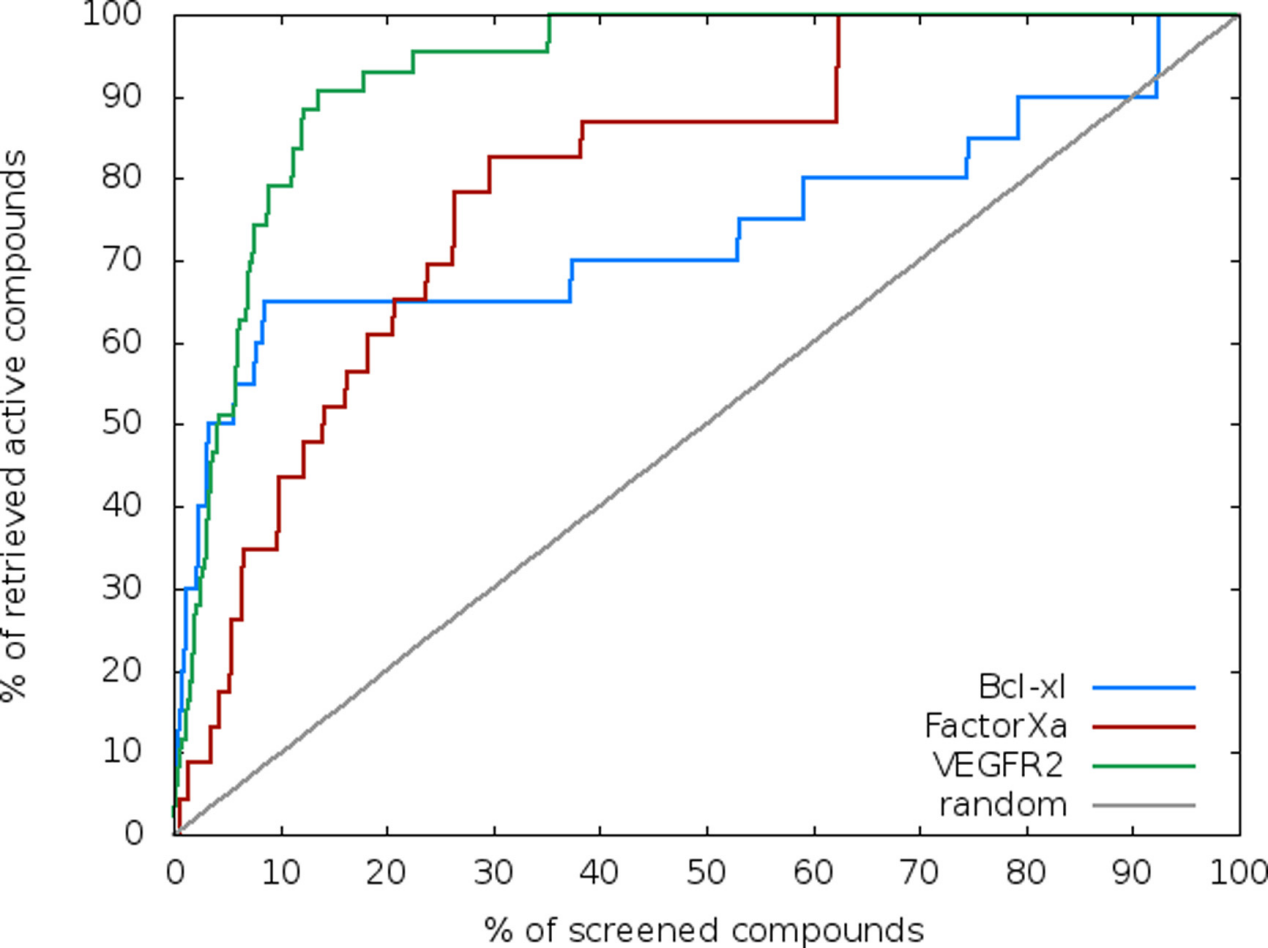
Enrichment curves of virtual screening of known actives and 1000 decoys performed with MTiOpenScreen on three protein targets.

The ability of MTiOpenScreen to discriminate small-molecule binder from non-binder compounds has been assessed on two enzymes, the catalytic domains of the RTK VEGFR2 and the serine protease Coagulation Factor Xa (FXa), and on the PPI target Bcl-xL. Screening was performed on 23, 43 and 20 actives of FXa, VEGFR2, Bcl-xL, respectively, and 1000 drug-like decoy molecules (see Materials and Methods for details). The enrichment curves of virtual screening performed with MTiOpenScreen on FXa, VEGFR2 and Bcl-xL are shown in Figure 3. MTiOpenScreen achieved very good performance with 43%, 80% and 65% of known actives retrieved for FXa, VEGFR2 and Bcl-xL, respectively at 10% of the screened library.

Regarding the speed performance, MTiAutoDock executes the blind docking of 10 ligands in 8 min in average for a protein receptor requiring a grid of 150×150×150 points and a grid spacing of 0.375 Å (e.g. a globular protein containing ∼300 residues and a size <55 Å per side). MTiAutoDock takes in average 25 min for a protein receptor requiring a grid of 170×170×170 points and a grid spacing of 0.6 Å and up to 1 h for a protein receptor requiring 180×180×180 points and a grid spacing of 0.8 Å. The screening of 10 000 compounds in a binding site of dimensions 25×25×25 Å is performed by MTiOpenScreen in 1 h. MTiOpenScreen can treat ∼170 queries for screening of 10,000 small molecules in a binding site of 25×25×25 Å per week.

## MATERIALS AND METHODS

### Implementation

MTiOpenScreen is implemented in the RPBS’ Mobyle Portal (27,28) ensuring a centralized workspace for the end user (bookmarked results and parameters are stored on the server) and control programs’ execution on the server side (storage and resources quota, jobs tracking, etc.). All jobs are kept for 30 days. There is no login requirement and data are not shared with other users. The security policy of Mobyle can be seen at http://mobyle.rpbs.univ-paris-diderot.fr/cgi-bin/portal.py#tutorials::Policy. Blind docking and binding site docking with MTiAutoDock employ the Lamarckian genetic algorithm (17) as implemented in AutoDock 4.2.6 to generate orientations/conformations of the compound. Ten docking runs are performed, with an initial population of 150 random individuals and a maximum number of 2 500 000 energy evaluations. We limit the maximum allowed grid dimensions for MTiAutoDock to 200×200×200 with grid resolution of 0.375 Å. The grid resolution is automatically replaced by 0.6 or 0.8 Å in cases where 0.375 Å is not sufficient to enter the entire protein receptor. Due to computational time limitations MTiOpenDocking cannot treat protein receptors bigger than 160 Å per side. In the case of binding site docking the grid dimensions and center are provided by the user or are automatically calculated based on the list of protein residues of the binding site uploaded by the user.

MTiOpenScreen performs virtual screening using docking with AutoDock Vina. AutoDock Vina (18) employs a gradient-based conformational search approach and defines the search space by a grid box defined by the box center coordinates and its dimensions of x, y and z. In AutoDock Vina the grid resolution is internally assigned to 1 Å. We use the number of binding modes of 10 and exhaustiveness of 8. The grid dimensions and center should be uploaded by the user or can be automatically calculated based on the list of protein residues of the binding site provided by the user. The scoring of the generated docking poses and ranking of the ligands is based on the Vina empirical scoring function approximating the binding affinity in kcal/mol.

### Chemical compound collections preparation

We prepared two electronic drug-like chemical libraries: a diverse chemical compound collection (Diverse-lib) and a focused chemical compound collection (iPPI-lib), the latter is tuned to target PPIs. We downloaded 12 chemical libraries from the PubChem BioAssay Database (19), assembling 3 574 650 molecules. After removing the redundant molecules, we employed an in-house developed ‘soft’ drug-like filter using the FAF-Drugs3 web server (24) to remove molecules with undesired physico-chemical properties (drug-likeness parameters used are given in Supplementary data). High-risk toxic groups and PAINS (29) as defined in FAF-Drugs3 were removed (more information on toxic groups and PAINS can be seen at http://fafdrugs2.mti.univ-paris-diderot.fr/groups.html). To ensure chemical diversity, the filtered drug-like 384 372 molecules were then clustered using the Cluster Molecule Protocol (Accelrys Pipeline Pilot v8.5) with the FCFP_4 fingerprint using a maximum distance of Tanimoto of 0.3 in the clusters. Finally, we retained the cluster centroids i.e. 99 288 diverse drug-like PubChem molecules, which constitute our diverse compound collection Diverse-lib.

Then, we generated a second chemical collection focused to target PPI. It has been recently observed that small PPI hit molecules often have physico-chemical characteristics slightly outside of what would be expected for a typical oral drug starting point (21,23,30–32). In order to prepare a collection enriched in low molecular weight modulators of PPI while remaining drug-like, we took the initial drug-like compound collection containing 384.372 PubChem molecules. We then used our in-house PPI-HitProfiler (31) to select PPI-friendly compounds. The program PPI-HitProfiler is based on a machine-learning model that was previously trained on 66 chemically diverse low MW inhibitors of PPI. The model uses a decision tree to select putative inhibitors of PPI by combining two properties, a shape descriptor and the unsaturation index (i.e. a property that can be related to a critical number of multiple bonds), and outputs the selected compounds into a focused chemical library. The remaining 204 728 molecules were then clustered using the Cluster Molecule Protocol (Accelrys Pipeline Pilot v8.5) with the FCFP_4 fingerprint using a maximum distance of Tanimoto of 0.3 in the clusters. This resulted in 51 232 drug-like molecules in the final iPPI-lib.

The 3D structures of the two predefined collections Diverse-lib and iPPI-lib were generated using the freely available web-server Frog2 (25). The procedure was launched keeping a maximum of one stereoisomer per compound without generating multiple ring conformations. The molecules were finally protonated at pH 7 using the major macrospecies option of the ChemAxon (www.chemaxon.com) calculator plugins.

### Preparation of data for performance assessment

All protein structures used to validate MTiOpenDocking and MTiOpenScreening were uploaded in PDB format. The PDB structures were cleaned and preprocessed automatically: all HETATMs were removed and hydrogen atoms were added to the structure using MGLTools (17). The grid center and dimensions of binding sites were defined using AutoDockTools4 (17) and then uploaded to the web server interface of MTiOpenDocking and MTiOpenScreening.

Ligands used to validate docking with MTiOpenDocking were extracted from PDB files (the list of PDB IDs is given in Supplementary data). Active compounds of FXa and VEGFR2 used to validate screening with MTiOpenScreening were taken from the PubChem BioAssay Database (see Supplementary data). Active compounds for Bcl-xL were taken from the database of PPI inhibitors iPPI-DB (33) (www.ippidb.cdithem.fr/) using ‘standard users access’. All active compounds were then filtered for drug-likeness using the software FAF-Drugs3 (24) and the same physico-chemical property ranges used for the Diverse-lib and iPPI-lib preparation. This resulted in 23, 43 and 20 actives for FXa, VEGFR2, Bcl-xL, respectively. The Diverse-lib and iPPI-lib libraries were used to randomly choose 1000 diverse decoy compounds to screen the two catalytic sites of FXa and VEGFR2, and 1000 PPI-friendly diverse molecules to screen the PPI target Bcl-xL. The 3D structures of the co-crystallized ligands and active compounds were generated using Frog2 (25) and were finally protonated at pH 7 using the major macrospecies option of the ChemAxon calculator plugins.

## CONCLUSION

The MTiOpenScreen web server aims at providing the scientific community with a free and user-friendly ligand docking and virtual screening tool. MTiOpenScreen offers the possibility to users to screen their own chemical libraries. It ensures automatic setup for the selected protein and two chemical libraries are provided for screening, Diverse-lib and iPPI-lib. The docking tool MTiAutoDock performing blind and binding site docking showed very good success rates: the right binding pocket was identified for 24 out of the 27 assessed proteins using blind docking and the top scored poses were found within an RMSD of 2.5 Å to the crystal structures for 60% of all tested complexes, and for 37% of them, the correct binding pose (RMSD < 1.5 Å) was found within the three most favorable ones when using binding site docking. MTiOpenScreen retrieved between 40% and 80% of knows actives for three different protein targets at 10% of the screened libraries. We believe that MTiOpenScreen can contribute to various chemical biology projects and to facilitate drug discovery particularly in academia.

## SUPPLEMENTARY DATA

Supplementary Data are available at NAR Online.

SUPPLEMENTARY DATA
