# Supplementary material for: MTiOpenScreen: a web server for structure-based virtual screening
Source: Nucleic Acids Res. 2015 Apr 8;43(Web Server issue):W448–54. doi: 10.1093/nar/gkv306 (PMC4489289; doi:10.1093/nar/gkv306)
Supplement: SUPPLEMENTARY DATA [file supp_43_W1_W448__index.html]

MTiOpenScreen: a web server for structure-based virtual screening — MTiOpenScreen: a web server for structure-based virtual screening — SUPPLEMENTARY DATA 

# MTiOpenScreen: a web server for structure-based virtual screening

## SUPPLEMENTARY DATA

**Files in this Data Supplement:**

- SUPPLEMENTARY DATA
- SUPPLEMENTARY DATA
