# Supplementary material for: MTiOpenScreen: a web server for structure-based virtual screening
Source: Nucleic Acids Res. 2015 Apr 8;43(Web Server issue):W448–54. doi: 10.1093/nar/gkv306 (PMC4489289; doi:10.1093/nar/gkv306)
Supplement: SUPPLEMENTARY DATA [file supp_gkv306_nar-00423-web-b-2015-File006.docx]

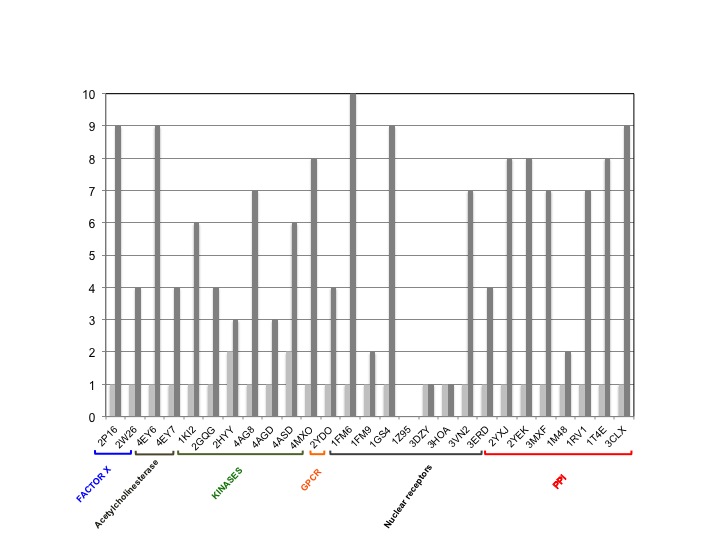


**Figure S1.** Ligand binding pocket identified by blind docking with MTiAutoDock. The number of poses among the 10 generated ones that found the right binding pocket are shown in black. The positions of the ranked pose that firstly identified the right binding pocket are shown in grey.
