# Supplementary material for: MTiOpenScreen: a web server for structure-based virtual screening
Source: Nucleic Acids Res. 2015 Apr 8;43(Web Server issue):W448–54. doi: 10.1093/nar/gkv306 (PMC4489289; doi:10.1093/nar/gkv306)
Supplement: SUPPLEMENTARY DATA [file supp_gkv306_nar-00423-web-b-2015-File007.docx]

**SUPPLEMENTARY DATA**

**Downloaded 12 chemical libraries from PubChem BioAssay Database to prepare the Diverse-lib and iPPI-lib collections:**

Burnham (443.033 compounds), Columbia (197,177 compounds), Emory (370,189 compounds), Johns Hopkins (354,331 compounds), NCGC (500,000 compounds), NMMLSC (388,672 compounds), PCMD (226,345 compounds), Pittsburgh (224,004 compounds), SRMLSC (226,666 compounds), The Scripps (417,966 compounds), UCLA (1395 compounds) and Vanderbilt (224,872 compounds).

**Active compounds for FXa and VEGFR2 used for virtual screening taken from the PubChem BioAssay Database:** (AID for VEGFR2: 259704, 263455, 614640, 614641, 614642, 614643, 718209, 761283; AID for FXa: 267984, 667405, 731259, 753178).

**Drug-like filter protocol:** We filtered compounds in the ranges: 100 < MW (Molecular Weight) < 600; 0 < tPSA (topological polar surface area) < 180; -3< logP< 6; 0< number of HBD (hydrogen bond donors) < 5; 0< number of HBA (hydrogen bond acceptors) < 12; 0< Rotatable Bonds< 11 ; 0< Rigid Bonds< 30 ; Num Rings ≤ 6 ; Max Size Ring ≤ 18 ; 3 < Num Carbon Atoms < 35 ; 1 < Num HeteroAtoms < 15 ; 0.1 < Ratio H/C < 1.1 ; Num Charges ≤ 3 ; -2 < Total Charge < 2.

**Protein-ligand complexes used for the validation of docking accuracy with MTiOpenDocking:**

*Protein-drug complexes PDB ID:* kinases: 2gqg, 2hyy, 1ki2, 4ag8, 4asd, 4agd, 4mxo; serine proteases: 2w26, 2p16; acetylcholinesterase: 4ey6, 4ey7; GPCR: 2ydo; nuclear receptors: 1gs4, 1z95, 3dzy, 1fm6, 1fm9, 3vn2, 3h0a, 3erd.

*PPI targets:* Bcl-xL in complex with ABT-737, PDB ID 2yxj; XIAP BIR3 domain in complex with Smac005, PDB ID 3clx; MDM2 in complex with an imidazoline and a benzodiazepine inhibitor, PDB ID 1rv1, 1t4e; interleukin-2 in complex with FRG, PDB ID 1m48; bromodomain BRD2 in complex with GSK525762, PDB ID 2yek; bromodomain BRD4 in complex with JQ1, PDB ID 3mxf.

**Protein structures used for the validation of virtual screening with MTiOpenScreen:**

*Factor Xa in complex with apixaban, PDB ID 2p16*

Grid center: x=7.446 y=42.63 z=60.722

Grid dimension: x=21 y=23 z=23

*VEGFR2 kinase domain in complex with axitinib, PDB ID 4ag8*

Grid center: x=20.192 y=25.726 z=35.76

Grid dimension: x=19 y=13 z=18

*Bcl-xL in complex with ABT-737, PDB ID 2yxj*

Grid center: x=-10.753 y=-39.086 z=-29.086

Grid dimension: x=22 y=14 z=29
